# What We Owe to Experiments on Animals

**Published:** 1904-01-09

**Authors:** Stephen Paget


### Hospital Clinics and Medical Progress.

### WHAT WE OWE TO EXPERIMENTS ON ANIMALS.

By Stephen Paget.

### *II.—PATHOLOGY, BACTERIOLOGY, AND THERAPEUTICS.*

(*Continued from page* 244.)

#### V.—Tetanus.

The old false theory, that tetanus (lock-jaw) was due to acute inflammation going up along an injured nerve to the brain, was destroyed by the experiments of Sternberg, Carle and Rattone, and Nicolaier (1880–1884), who proved, by experiments on animals, that tetanus is due to a specific organism which abounds in the superficial layers of the soil, in garden earth, and in dust. This organism, introduced under the skin by a graze, or a rusty nail, or a splinter of wood, flourishes there; and its chemical products in the blood, like a dose of strychnine, have a direct action on the higher nerve-centres, causing convulsions and often death. Among horses, tetanus is not rare, and in some parts of the world it is terribly common among men:—

"Tetanus is an exceedingly common disease in some tropical countries. In Western Africa, for example, a large proportion of wounds, no matter how trifling as wounds they may be, if they are fouled by earth or dirt, result in tetanus. The French in Senegambia have found this to their cost. . . In many countries, so general and so extensive is the distribution of the tetanus bacillus that trismus neonatorum is a principal cause of the excessive infant mortality." (Mabson, "Tropical Diseases.")

The study of the disease, from 1884 onward, was beset by a thousand difficulties, which cannot be put here. But, by 1894, Behring and Kitasato were able to immunise animals against it: and, in 1895, the first cases treated with an antitoxin were reported—42 cases with 27 recoveries. Unhappily, the first sign of the disease is the last stage of it; there is no warning, no thought of the truth, till the highest nerve-centres are suddenly attacked. In acute cases, neither antitoxin, nor anything else, nor everything else with or without antitoxin, can offer any good hope of recovery. In cases that are less acute, the patient may hold on till he has brewed his own antitoxin out of his own blood, and so may recover "of himself." Among acute cases, the injection saves lives here and there, but no great multitude of them: among the non-acute cases, recovery may be due not so much to the treatment as to nature. Perhaps the most that can be said here is:—

1. The antitoxin does not interfere in any way with other methods of treatment: it can be added to them, not used instead of them.

2. Dr. Lambert's monograph on the subject (*New York Medical News*, July, 1900), a very careful and dispassionate study of 279 antitoxin cases, says:—"Taking the statistics as a whole, there is a distinct improvement in the mortality of tetanus since the introduction of antitoxin."

3. The opponents of all experiments on animals praise Baccelli's treatment of tetanus without antitoxin. They forget that Baccelli's treatment is the direct result of Nicolaier's experiments. And so will every treatment be, of all the treatments that we shall ever live to see. Heaven send us a better drug than we have got at present; but when it comes, it will come as it were from Nicolaier's hands.

4. Beside the general treatment, there is also the local treatment of the original wound. Bacteriology has explained, and brought into general acceptance, the rule that the wounded tissues should be freely and immediately excised, wherever this is practicable.

Let us now consider not the curative value, but the *protective* or *preventive* value, of tetanus-antitoxin. Horses can be immunised, by a dose of it, against the disease. Here are two instances:—

1. *France.*—"The results of Nocard's method of preventive inoculations in veterinary practice are most striking. Among 63 veterinarians, there have been inoculated 2,737 animals with preventive doses of antitoxin, and not a single case of tetanus developed; while *during the same period, in the same neighbourhoods*, 259 cases of tetanus developed in non-inoculated animals." (*Medical News*, July 7, 1900).

2. *United States.*—"Studies were made upon several hundred horses used for the production of various serums in one of the large laboratories of the United States. The horses, because of the constant manipulations, frequently became infected with tetanus, and in 1897 and 1898, when scrupulous cleanliness and disinfection were the only precautions employed to prevent the disease, the death-rate varied from 8 to 10 per cent. During 1899, nearly 200 horses were subjected to systematic immunisation with tetanus-antitoxin; and, *in spite of otherwise similar conditions*, the death-rate descended to 1 per cent." (*Med. Annual*, 1901.)

And, among ourselves, there are risks, happily not common, that might well be met by a protecting dose of the antitoxin. A bacteriologist might cut his hand by breaking a test-tube containing a tetanus culture; a traveller in the Congo territory might receive a poisoned arrow wound; a groom might tear his hand in a stable where a horse had died of the disease. These instances may seem fanciful; but there are facts also to be had—the cases at the Gebaer Anstalt at Prague (1899), the cases in the jute mills at Dundee (1900), and the curious "Fourth of July" cases. American boys, on that day, love the firing of toy-pistols, with wads made of scraps of paper picked off the streets. The pistol bursts, or goes off prematurely, and the wad, bacilli and all, is lodged under the skin of the hand, and out of reach of home surgery. In 1899, after the Fourth of July, 83 cases of tetanus were reported, 26 of them in or around New York. It is no wonder that an American surgeon has recommended a protective dose of the antitoxin after the occurence of this particular wound. (See *Med. News*, June 1st, 1901.)

(*To be continued.*)

